# Hepatitis A Outbreak Among Adults with Developmental Disabilities in Group Homes — Michigan, 2013

**Published:** 2015-02-20

**Authors:** Susan R. Bohm, Keira Wickliffe Berger, Pamela B. Hackert, Richard Renas, Suzanne Brunette, Nicole Parker, Carolyn Padro, Anne Hocking, Mary Hedemark, Renai Edwards, Russell L. Bush, Yury Khudyakov, Noele P. Nelson, Eyasu H. Teshale

**Affiliations:** 1 Division of Communicable Disease, Michigan Department of Community Health; 2 Medical Services, Oakland County Health Division; 3 Communicable Disease Unit, Oakland County Health Division; 4 Disease Control, Macomb County Health Department; 5 Tuscola County Health Department; 6 Division of Viral Hepatitis, National Center for HIV/AIDS, Hepaitis, STD, and TB Prevention, CDC

Hepatitis A virus (HAV) infections among persons with developmental disabilities living in institutions were common in the past, but with improvements in care and fewer persons institutionalized, the number of HAV infections has declined in these institutions (1). However, residents in institutions are still vulnerable if they have not been vaccinated. On April 24, 2013, a resident of a group home (GH) for adults with disabilities in southeast Michigan (GH-A) was diagnosed with hepatitis A and died 2 days later of fulminant liver failure. Four weeks later, a second GH-A resident was diagnosed with hepatitis A. None of the GH-A residents or staff had been vaccinated against hepatitis A. Over the next 3 months, six more cases of hepatitis A were diagnosed in residents in four other Michigan GHs. Three local health departments were involved in case investigation and management, including administration of postexposure prophylaxis (PEP). Serum specimens from seven cases were found to have an identical strain of HAV genotype 1A. This report describes the outbreak investigation, the challenges of timely delivery of PEP for hepatitis A, and the need for preexposure vaccination against hepatitis A for adults living or working in GHs for the disabled.

The Michigan Department of Human Services licenses approximately 200 and 170 GHs for adults with developmental disabilities in Oakland and Macomb Counties, respectively. GHs, owned and operated by various companies, provide 24-hour care and supervision for up to six residents, who share rooms and bathrooms. Residents have developmental and/or physical disabilities; some are nonverbal or minimally communicative, and some require assistance with toileting. The average staff-to-resident ratio is 2:1. Residents attend various programs at off-site work sites (WSs) including vocational centers for the disabled, a restaurant, and hotel, where they have contact with off-site workers and residents from other GHs or private homes.

After the hepatitis A diagnosis in a GH-A resident, the Oakland County Health Division (OCHD) began an investigation on April 24, 2013, to identify the source of infection and to prevent HAV transmission to other residents and staff. For purposes of this investigation, a confirmed case of hepatitis A was a case meeting the Council of State and Territorial Epidemiologists case definition for acute hepatitis A (http://wwwn.cdc.gov/nndss) in a person who resided or worked at an adult GH or WS during April 16–September 18, 2013.

A second case in GH-A was diagnosed May 16, 2013, in a person who attended WS-A (Table 1). At a second Oakland County group home (GH-B), three cases were reported among residents, with illness onset dates of May 17 (case 3), May 28 (case 5), and May 29 (case 6) (Figure 1). Case 4, in a resident of GH-C who attended WS-B in Macomb County, was diagnosed on May 26, 2013. Patients 3 and 4 had no previous contact with patients 1 or 2. Patients 3 and 4 attended WS-B, and patients 5 and 6 attended WS-C and WS-D, respectively. A health care worker (HCW-1), who was employed at GH-A, GH-B, and WS-B, was identified as a common link for the first six cases. HCW-1 did not report any symptoms and had not previously received the hepatitis A vaccine. Five cases were in residents of the GHs where HCW-1 worked, and HCW-1 cared for patient 4 while at WS-B (Figure 2).

At a fourth GH (GH-D) in Oakland County, patient 7 became symptomatic on July 5, 2013. A fifth GH (GH-E) reported case 8, in a resident with an approximate illness onset date of July 23, 2013. Patients 7 and 8 had no direct contact with any previous patient, nor did they attend the same WS. However, two GH-D residents (one was the roommate of case 7) attended WS-B where they were likely exposed to patients 3 or 4 or both. Two GH-E residents worked at WS-D, the same vocational center that patient 6 attended. Patient 8 attended a special needs camp in Tuscola County during June 30–July 12, 2013. The camp and Tuscola County Health Department were notified on July 25, 2013, of the potential exposure to other campers and camp staff.

Eight GH residents in five adult GHs in Oakland and Macomb Counties developed hepatitis A (Table 1). None of the residents and only eight (14%) of 57 HCWs in the five group homes had previously received hepatitis A vaccine. Illness onset dates ranged from April 16 to July 23, 2013. Ages of patients ranged from 42 to 61 years, with an average age of 48 years (median age = 48 years). Seven of the eight patients were male; three of the five homes housed only males. GH attack rates, calculated as the number of cases per home divided by the number of susceptible GH residents, ranged from 16.7% to 60.0% among the five homes; the attack rate among susceptible residents in all five homes was 27.6%. At GH-A and GH-B, where HCW-1 worked, the attack rates were 33.3% and 60.0%, respectively. After the occurrence of case 8, no further hepatitis A cases were detected among contacts.

No common food source was identified among the five group homes. Because a multistate hepatitis A outbreak was occurring concurrently that implicated a frozen berry product, GH managers were asked if frozen berries were consumed; none were. No staff reported any symptoms or previous diagnosis with hepatitis A. It was noted that HCW-1 did not always use gloves when assisting residents with toileting. Serum from seven of the eight patients was found to have the same HAV genotype 1A strain, sharing the identical VP1/P2B genomic sequence.

Of the 261 contacts who warranted PEP, 225 (86.2%) were confirmed to have received immunoglobulin (IG) or hepatitis A vaccine or both (Table 2).

## Discussion

Since 2006, the Advisory Committee on Immunization Practices (ACIP) has recommended routine vaccination against hepatitis A for all children at age 1 year (1), but its current recommendations for adults do not include residence in an institution or GH as an indication for vaccine (3). The 2006 ACIP recommendations note that in the past, HAV infection has been highly endemic in institutions for persons with developmental disabilities (1). However, because fewer persons have been institutionalized and conditions in institutions have improved, the incidence and prevalence of HAV infection have decreased, although outbreaks can occur in these settings (1). Disabled adults are now typically cared for in group homes, where residents live in close quarters and are often incontinent and nonverbal. These factors, as well as lack of contact precautions and hand washing might have contributed to the spread of HAV in this outbreak, similar to how transmission among diapered children in daycare settings was linked to community outbreaks of HAV infection during the prevaccine era (1). Moreover, after the introduction of hepatitis A vaccine in 1996, the age-specific patterns of disease have shifted to include an increasing proportion of susceptible adolescents and adults because of less exposure to infected children (4). Thus, the unvaccinated adult population in group homes is at high risk for HAV infection.

ACIP hepatitis A vaccine PEP recommendations were followed in this outbreak (2). Vaccine was used if IG was not available. Local public health workers partnered with two hospitals at clinics set up to provide PEP. Public health workers also administered vaccine at county clinics and at WS-F. Some staff and residents received PEP from their health care providers. OCHD notified neighboring counties of potential exposures in residents of GHs or in attendees at WSs within their respective counties. Vaccinations of contacts were verified through state and county immunization databases, or OCHD followed up with providers to confirm that PEP had been administered.

What is already known on this topic?

Hepatitis A virus (HAV) infections among persons with developmental disabilities living in institutions were common in the past. With improvements in care and fewer persons institutionalized, the number of HAV infections has declined in these institutions. However, residents in institutions are still vulnerable if they have not been vaccinated.

What is added by this report?

During April–July 2013, eight residents of five group homes for adults with disabilities in Michigan were diagnosed with hepatitis A, and one died; none had been vaccinated against hepatitis A. Serum from seven of the eight was found to have HAV genotype IA strain, sharing the identical VP1/P2B genomic sequence. Of the 261 contacts who warranted postexposure prophylaxis, 86.2% received either the recommended immunoglobulin, hepatitis A vaccine, or both.

What are the implications for public health practice?

This outbreak report highlights the risk for HAV infection among adults living or working in small group home settings for the disabled and the public health resources needed to respond to outbreaks in these settings. A public health response plan for hepatitis A outbreaks should include pre-identification of sources of immunoglobulin and hepatitis A vaccine. Routine vaccination of residents and staff of the group homes might have prevented this outbreak and the costs of containing it.

Although ACIP recommendations were followed in this outbreak, PEP administration was not without challenges. In Michigan, local health departments are responsible for having a hepatitis A outbreak response plan that pre-identifies sources of hepatitis A vaccine and IG in the community. In this outbreak, although on-hand IG supplies at the pre-identified hospitals expedited administration of PEP, they were not sufficient to provide PEP for such a large cohort. However, hospital pharmacies were able to respond quickly, order IG and vaccine, and received shipments overnight from their suppliers. It is likely that because of the rapid public health response this hepatitis A outbreak in group homes involved only five of 370 homes in the two affected counties. Before the outbreak, the estimated hepatitis A vaccination coverage rate among staff and residents in the affected homes was only 6%, which is thought to be typical of vaccination coverage in other homes in the two counties. Thus, the risk for substantial spread was high. This outbreak raises the question of whether adult residents and staff of group homes, in light of increasing adult susceptibility, should be considered a high risk group for HAV transmission and a group for whom pre-exposure vaccination should be recommended.

When case 8 was detected, the 14-day window for effective PEP had passed, and PEP was not recommended to campers and camp staff at the special needs camp. Tuscola County Health Department provided education information on July 26, 2013, for approximately 70 staff working at the camp. Campers who attended during June 30–July 12 were notified of their potential exposure and told to seek medical attention if they developed symptoms suggestive of HAV infection.

In the United States, the most common risk factor identified for HAV infection is travel (5). However, a risk factor for HAV infection is unknown in 35%–85% of U.S. cases, depending on the surveillance source (6). Similarly, the initial source of the infection in this outbreak has not been identified. However, given the multiple sites of employment and lack of hand hygiene of HCW-1, it is plausible that HCW-1 played a role in transmitting the virus among cases 1 to 6. The connection to the outbreak for cases 7 and 8 is more indirect. The two residents of GH-D and the two residents of GH-E, who had exposures to confirmed hepatitis A cases at WS-B and WS-D, respectively, might have introduced HAV into their group homes. Almost all (94%) residents and staff in the Michigan GHs and WSs were unvaccinated against hepatitis A and thus were susceptible.

Several public health actions were undertaken to prevent further transmission of HAV, including education on proper hand hygiene and glove use, cancellation of outings (overnight camp), and administration of PEP. Because the staff at the facilities had multiple roles, such as preparing food and assisting residents with their toileting, OCHD asked GHs to bring in outside staff for food preparation. Contacts (defined as co-residents and staff at five GHs and seven WSs who had direct contact with hepatitis A cases) were evaluated by local public health providers for PEP with IG, hepatitis A vaccine, or both.

The findings in this report are subject to at least two limitations. First, the immune status of residents and contacts before onset of this outbreak is not known. Second, only symptomatic cases of HAV infection were identified and diagnosed. Some residents with mild illness might not have been recognized because some of the residents are nonverbal.

This outbreak report highlights the risk for HAV infection among adults living or working in small group home settings for the disabled and the public health resources needed to respond to outbreaks in these settings. A public health response plan for hepatitis A outbreaks should include pre-identification of sources of IG and hepatitis A vaccine. Routine vaccination of residents and staff of the GHs might have prevented this outbreak and the costs of containing it.

## Figures and Tables

**FIGURE 1:**
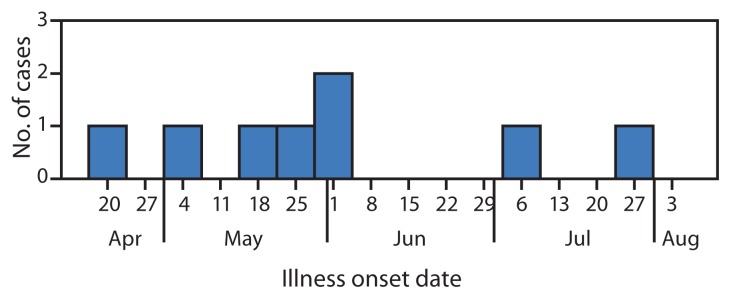
Number of laboratory-confirmed hepatitis A cases among residents of group homes, by illness onset date — Michigan, April–July 2013

**FIGURE 2:**
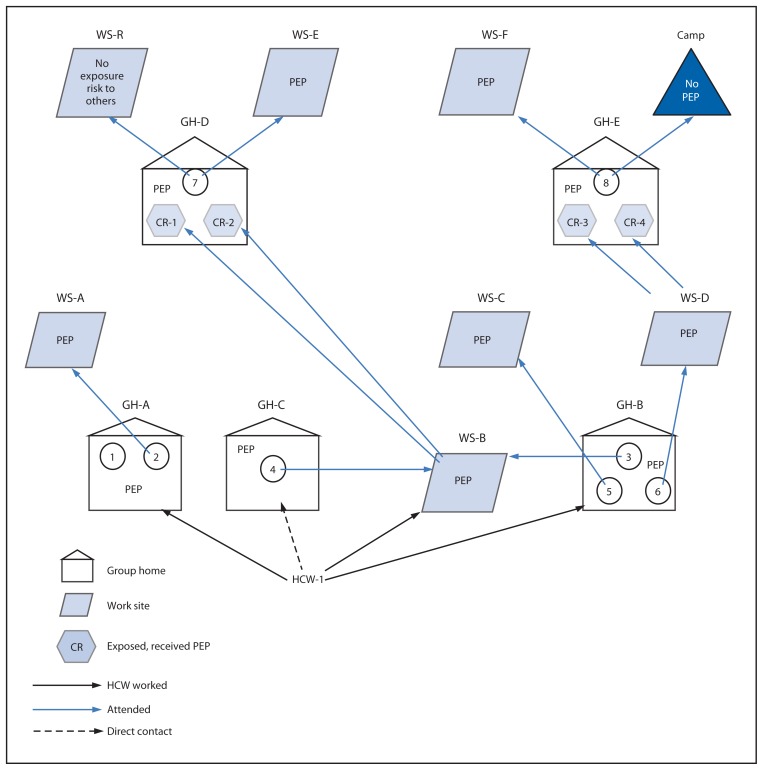
Schematic of the suspected route of transmission of hepatitis A virus among residents of group homes and their work sites — Michigan, April–July 2013 **Abbreviations:** GH = group home; WS = work site; PEP = postexposure prophylaxis; CR = co-resident; HCW = health care worker.

**TABLE 1:** Epidemiologic and clinical summary of hepatitis A cases among residents of group homes — Michigan, April–July 2013

Case no.	Group home	Work site	Age (yrs)	Sex	Illness onset date	Date of diagnosis	Clinical/Laboratory results	Symptoms
1	A	None	48	M	4/16	4/24	IgM anti-HAV+; ALT 2,525 U/dL; AST 4,530 U/dL	Yellow sclera, dark urine
2	A	A	45	M	5/4	5/16	IgM anti-HAV+; ALT 436 U/dL; AST 175 U/dL	Lethargic, chalky stools, dark urine, diarrhea, jaundice
3	B	B	47	M	5/17	5/21 by clinical laboratory; 5/31 confirmed by MDCH laboratory	IgM anti-HAV+; ALT 693 U/dL; AST 118 U/dL	Painless jaundice, no other symptoms
4	C	B	49	M	5/23	5/26	IgM anti-HAV+; ALT 1,314 U/dL; AST 463	Weak, difficulty standing
5	B	C	42	M	5/28	5/29	IgM anti-HAV+; ALT 4,946 U/dL; AST 4,521 U/dL	Jaundice, dark urine, decreased appetite
6	B	D	49	M	5/29	5/30	IgM anti-HAV+; ALT 1,419 U/dL; AST 792 U/dL	Abdominal pain, decreased appetite
7	D	E	45	M	7/5	7/10	IgM anti-HAV+; ALT 2,434 U/dL; AST 2,082 U/dL	Nausea, vomiting, orange urine, jaundice
8	E	F	61	F	7/23	7/25	IgM anti-HAV+; ALT 1,291 U/dL; AST 980 U/dL	Jaundice

**Abbreviations:** IgM = immunoglobulin M; HAV = hepatitis A virus; ALT = alanine aminotransferase; AST = aspartate aminotransferase; MDCH = Michigan Department of Community Health.

**TABLE 2:** Number of persons who received postexposure prophylaxis (PEP) and date of PEP among group home and work site contacts of persons with hepatitis A infection* — Michigan, April–July 2013

Case no.	Group home	Work site	No. of group home contacts	Group home contacts who received PEP		PEP dates at group homes	No. of work site contacts	Work site contacts who received PEP		PEP dates at work sites
	
				No.	(%)			No.	(%)	
1	A	None	18	18	(100)	Vaccine: 4/29–5/3, 5/15; IG: 5/17	N/A	N/A		N/A
2	A	A	18	18	(100)	Vaccine: 4/29–5/3, 5/15; IG: 5/17	12	8	(66.7)	6/10
3	B	B	19	13	(68.4)	5/31–6/6	55	53	(96.4)	5/31–6/6
4	C	B	14 (2 previously vaccinated)	14	(100)	6/3–6/24	55	53	(96.4)	5/31–6/6
5	B	C	19	13	(68.4)	5/31–6/6	4	4	(100)	6/5–6/11
6	B	D	19	13	(68.4)	5/31–6/6	57	46	(80.7)	6/1–6/17
7	D	E	13 (2 previously vaccinated)	13	(100)	7/11–7/15 (2 received PEP at work site on 6/6)	10	5	(50)	7/15–7/19
8	E	F	13 (1 previously vaccinated)	12	(92.3)	7/30–8/2 (2 received PEP at work site on 6/10)	45	39	(86.7)	7/29–7/31
		Camp	70 staff and 89 campers from group home and private residences in 16 counties in Michigan	None†	—	N/A	N/A	N/A	—	N/A

**Abbreviations:** IG = immunoglobulin; N/A = not applicable.

* Persons vaccinated against hepatitis A before the outbreak were not included among those needing PEP.

† 14-day period for effective PEP expired on day of notification about case. Campers were notified.
